# Lanthanoid-doped BiVO_4_ microswimmers with built-in photon upconversion and light-driven motion†

**DOI:** 10.1039/d5cc02091c

**Published:** 2025-07-08

**Authors:** João Marcos Gonçalves, Luisa Natalia Córdoba Urresti, Yufen Chen, Katherine Villa

**Affiliations:** a Institute of Chemical Research of Catalonia (ICIQ), The Barcelona Institute of Science and Technology (BIST) Av. Països Catalans, 16 Tarragona E-43007 Spain kvilla@iciq.es; b Departament de Química Física i Inorganica, Universitat Rovira i Virgili 43007 Tarragona Spain

## Abstract

We report a one-step synthesis of lanthanoid-doped BiVO_4_ microswimmers that integrate photon upconversion and light-driven propulsion within a single particle. Yb^3+^/Er^3+^ doping enables near-infrared-to-visible upconversion, while BiVO_4_ provides photocatalytic propulsion. This straightforward strategy avoids multi-component assembly and opens up new opportunities for designing multifunctional photoactive microswimmers with intrinsic luminescence for potential use in environmental and biomedical applications.

## Abstract 2

A single-step synthesis yields lanthanoid-doped BiVO_4_ microswimmers that combine visible-light-driven self-propulsion and near-infrared photon upconversion within a single material.

## Graphical abstract

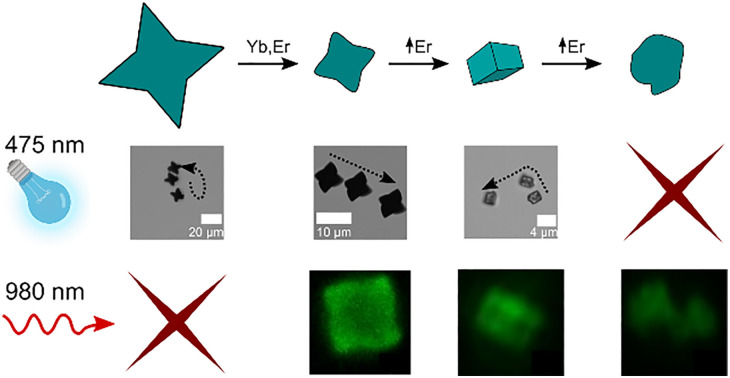

Artificial microswimmers (MSs), also commonly referred to as micromotors or microrobots, are micron-sized particles capable of self-propulsion when exposed to external stimuli such as electric and magnetic fields, ultrasonic waves, among others.^1^ Their active propulsion enhances interaction with the surrounding environment and allows them to overcome mass transfer limitations,^2,3^ making them attractive for applications such as targeted cargo delivery,^4^ bacterial and biofilm inactivation,^5–7^ and for traversing biological barriers.^8^ Among the available stimuli, light offers unique advantages such as easy manipulation and instrumentation, remote control and the possibility of using sunlight as an abundant power source.^9–11^ To expand the functionality and versatility of light-driven MSs, integrating additional optical features such as photon upconversion (UC) is particularly appealing.

UC is a non-linear optical phenomenon that converts low energy photons into higher energy ones by a multi-photon absorption process.^12^ This property has been widely exploited to extend the activity of photocatalysts under NIR light and to enable deep-tissue bioimaging by taking advantage of excitation within the biological transparency windows.^13–16^ Recently, UC nanoparticles have been coupled with MSs to enable functionalities such as real-time tracking, photodynamic therapy, and enhanced optoacoustic signals.^17,18^ However, these approaches rely on the integration of separately synthesized components, often requiring complex, multi-step fabrication. A single-material system combining upconversion and visible-light propulsion through a one-step synthesis would greatly simplify fabrication and broaden the application of multifunctional MSs in biomedical and environmental fields. BiVO_4_ emerges as a promising platform to unify these functionalities, as it has been widely studied both as a visible-light-driven microswimmer and as an upconversion host material.^19–21^ Herein, we report the one-step synthesis of lanthanoid-doped BiVO_4_ MSs that exhibit both photon upconversion and light-driven self-propulsion within a single particle. Doping with Yb^3+^ and Er^3+^ ions enables efficient upconversion under 980 nm excitation,^12^ while the BiVO_4_ matrix supports photocatalytic propulsion under visible light. This dual-functionality, achieved without the need for multi-component assembly, expands the practical potential of multifunctional photoactive MSs.

Aqueous solutions of Bi(NO_3_)_3_ and NaVO_3_ were mixed with KCl as a morphology-controlling additive. Cl^−^ ions reduce the surface energy of {010} facets, promoting shape modification, while potential BiOCl intermediate phases may further influence the particle morphology.^22–24^ The mixture was sonicated for 1 h, subjected to hydrothermal treatment (180 °C, 12 h), and doped with varying Yb^3+^ and Er^3+^ concentrations relative to Bi^3+^. The Yb^3+^ concentration was fixed at 20%, while the Er^3+^ content was adjusted. For simplicity, the samples are labelled according to their the Er^3+^ concentration only (see the ESI† for details).

The influence of lanthanoid doping on the MSs morphology and size was analyzed using field-emission scanning electron microscopy (FESEM) and energy-dispersive X-ray spectroscopy (EDS) mapping (Fig. 1a–d). Pristine BiVO_4_ MSs exhibited a four-point star-shaped morphology with an average size of 18 ± 3 μm, consistent with previous studies.^22,25^ EDS mapping confirmed uniform distributions of Bi, V, and O (Fig. 1a). Doping with 15%Er^3+^ preserved the star-like shape but reduced the average size to 8 ± 1 μm (Fig. 1b). At 18%Er^3+^, the MSs further decreased in size (2.8 ± 0.5 μm) and transitioned to square prismatic structures (Fig. 1c). For 20%Er^3+^, the morphologies became less defined, though the size continued to diminish (Fig. 1d). This progressive size reduction is summarized in Fig. 1e and Fig. S1, S2 (ESI†), demonstrating a clear correlation with the Er^3+^ concentration. Despite these morphological changes, EDS mapping verified homogeneous elemental distributions (Bi, V, Yb, and Er) in all samples, confirming successful doping.

**Fig. 1:**
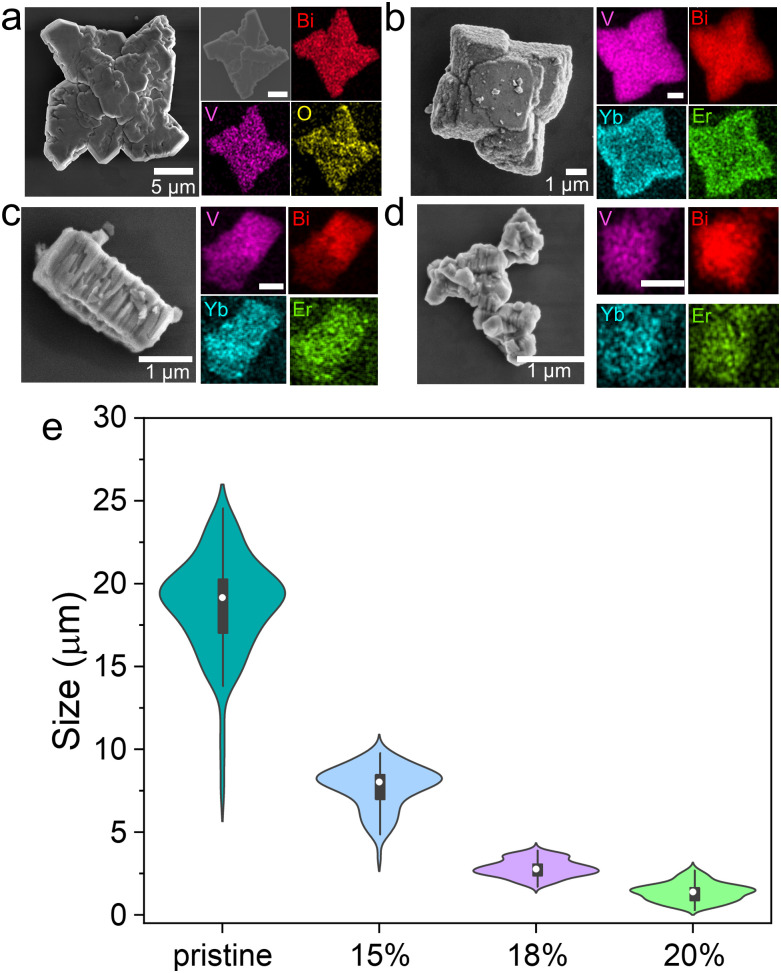
Morphological characterization of BiVO_4_-based MSs. FESEM images, EDS mapping and size distribution of doped samples. (a) Pristine, (b) 15%Er^3+^, (c) 18%Er^3+^ and (d) 20%Er^3+^. (e) Size distribution plot of all MSs.

The crystalline phases were analysed by X-ray diffraction (XRD), as shown in Fig. S3 (ESI†). Pristine BiVO_4_ (undoped) exhibits a pure monoclinic scheelite structure, known to be more photocatalytically active than its tetragonal counterpart.^26^ Upon lanthanoid doping, however, a mixed-phase composition emerges due to the tendency of these dopants to favour tetragonal phase formation.^19^ Additionally, small impurities corresponding to the formation of ErVO_4_ were also detected, which increased with higher dopant concentration. Table S1 (ESI†) presents semi-quantitative phase analysis results obtained according to the relative peak intensity of each phase, revealing a clear increase in the tetragonal content with higher dopant concentrations, as well as the secondary ErVO_4_ phase. The observed decrease in the size and changes in the morphology might be attributed to the contribution of these additional phases.

The optical absorption properties of the MSs are shown in Fig. 2. The bandgap energies of the samples were estimated using Tauc plot analysis (Fig. 2a). Pristine BiVO_4_ exhibited a bandgap of 2.34 eV, which agrees with the values reported in the literature for BiVO_4_.^27,28^ An increase in the dopant concentration resulted in a progressive blue shift in the bandgap, likely due to the coexistence of monoclinic and tetragonal phases, which tend to widen the bandgap.^19,20^ While the calculated bandgap for the 18%Er^3+^-doped sample was 2.84 eV, a residual absorption feature was observed between 450 and 500 nm, indicating potential excitability under 475 nm irradiation. This behavior is expected to have implications for the self-propulsion of these MSs. Two additional absorption bands were observed in the Er^3+^-doped samples (Fig. S4, ESI†), corresponding to the characteristic Er^3+^ transitions (^2^H_11/2_ ← ^4^I_15/2_ around 520 nm and ^4^F_9/2_ ← ^4^I_15/2_ around 650 nm). To avoid interference from the material bandgap and facilitate visualization, a baseline correction was performed (Fig. 2b and Fig. S5, ESI†). The corrected spectra reveal a clear increase in the 650 nm band intensity with increasing Er^3+^ content, with the 20%Er^3+^ sample showing the highest absorption (Fig. S5, ESI†). Overall, the emergence and evolution of these Er^3+^-specific transitions confirm the successful incorporation of Er^3+^ ions into the BiVO_4_ MSs (Fig. 2b). While the absorption peak at 650 nm was notably weaker than the prominent peak at 525 nm (Fig. S5, ESI†), its intensity increased consistently with higher Er^3+^ content. Although intermediate doping concentrations produced similar absorption profiles, the highest doping level led to a significant increase in absorption intensity. This, togetherwith the data shown in Fig. 2b, demonstrates the successful incorporation of Er^3+^ into the host matrix.

**Fig. 2:**
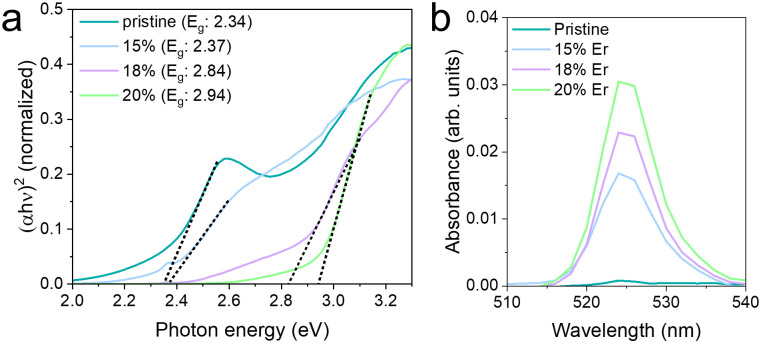
Optical characterization of BiVO_4_-based MSs. (a) Tauc plot of the MSs and their respective bandgap and (b) absorbance in the 510–540 nm region, showcasing the increase in the absorption attributed to Er^3+^.

The introduction of Yb^3+^–Er^3+^ dopant pair imparts UC emission properties to the as-synthesized BiVO_4_ MSs, as shown in Fig. 3a. This emission arises from energy transfer upconversion (ETU), where Yb^3+^ absorbs NIR photons and transfers the energy to Er^3+^, which then emits visible photons, as shown in the energy level diagram in Fig. S6 (ESI†).^12,29^ Under 980 nm excitation, the emission spectrum presents characteristic Er^3+^ transitions: (^2^H_11/2_, ^4^S_3/2_) → ^4^I_15/2_ between 500 and 550 nm and ^4^F_9/2_ → ^4^I_15/2_ around 675 nm.^29,30^ Interestingly, this visible emission upon NIR excitation enables fluorescence imaging of the MSs, as presented in Fig. 3b–d. As reported in the literature, lanthanoid-doped BiVO_4_ exhibits low cytotoxicity,^20^ making NIR excitation particularly advantageous for biomedical applications due to its superior tissue penetration depth and reduced scattering compared to visible light.

**Fig. 3:**
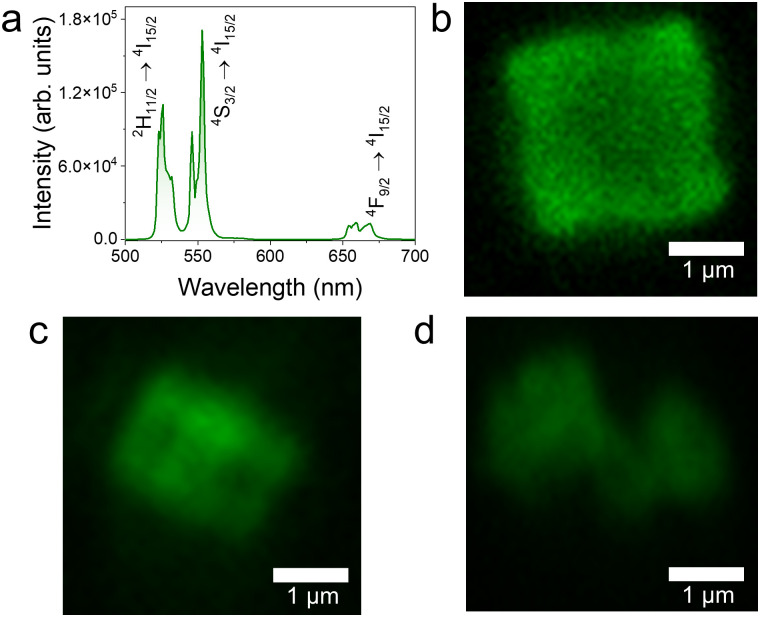
Upconversion characterization of lantanoid-doped BiVO_4_ MSs. (a) UC emission of the 15%Er MSs and fluorescence images of (b) 15%Er^3+^, (c) 18%Er^3+^ and (d) 20%Er^3+^ (*λ*_exc_ = 980 nm; power density = 66.2 W cm^−2^).

The number of photons involved in the UC process was estimated using the intensity power law *I* ∝ *P*^*n*^, where *I* is the emission intensity, *P* is the excitation power density and *n* is the number of photons (Fig. 4).^31^ Regardless of the Er^3+^ concentration, the number of photons was determined to be 2, in agreement with the literature.^19^ This confirms a typical UC mechanism where Yb^3+^ absorbs two NIR photons and transfers the energy to Er^3+^, which then emits visible photons.

**Fig. 4:**
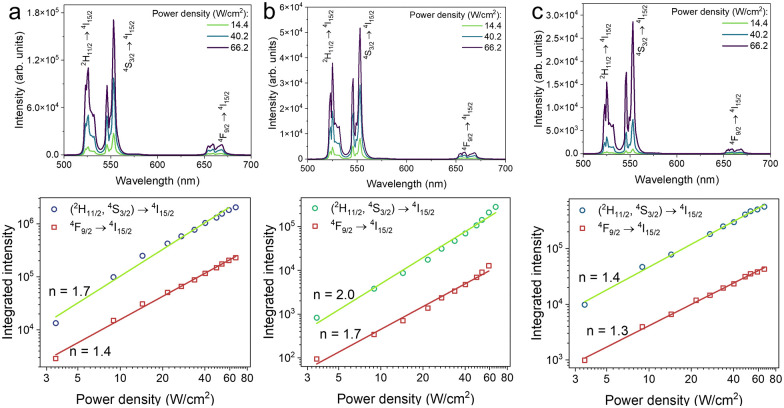
Upconversion intensity dependence on excitation power density (top panel) to estimate the number of photons involved in the process using the intensity power law (bottom panel). (a) 15%Er^3+^, (b) 18%Er^3+^ and (c) 20%Er^3+^.

To test self-propulsion, BiVO_4_-based MSs were dispersed in a hydroquinone/benzoquinone (HQ/BQ, 50 mM/5 mM) solution and monitored their motion behaviour in the absence of light (Video S1, ESI†). Under these conditions, all particles displayed random trajectories consistent with Brownian diffusion. Upon 475 nm light exposure, the pristine, 15%Er^3+^-, and 18%Er^3+^-doped BiVO_4_ MSs displayed autonomous propulsion (Video S2, ESI†), confirming their photoactivation. In contrast, the 20%Er^3+^-doped sample did not exhibit any motion under blue light, a behavior attributed to its higher bandgap and lack of absorption in this spectral region. Although the effect of light intensity was not examined in detail, active propulsion was observed at intensities as low as 367 mW cm^−2^ for the pristine sample, 437 mW cm^−2^ for the 15%Er^3+^-doped sample and 565 mW cm^−2^ for the 18%Er^3+^-doped sample (Video S3, ESI†).

The observed propulsion behaviour is mediated by the photoactivation of BiVO_4_, whereby incident photons generate electron–hole pairs that trigger surface redox reactions,^9^ producing an uneven distribution of chemical species on the surface of the microswimmer and initiating phoretic self-propulsion.^32^ In addition, the heterogeneous surface composition and anisotropic morphology further enhance the asymmetry of the system, reinforcing the conditions necessary for directed motion. More details on the propulsion mechanism are presented in the ESI.† ^21,33,34^

Representative trajectories for the pristine, 15%Er^3+^ and 18%Er^3+^ MSs are shown in the top panels of Fig. 5a–c, respectively, confirming that self-propulsion is retained even with significant lanthanoid doping. Additional trajectories are presented in Fig. S7 (ESI†). Mean square displacement (MSD) plots (bottom panels of Fig. 5a–c) further confirm the transition from linear to quadratic behaviour under visible light illumination, indicating active propulsion.^35^ Comparison of the trajectories in Fig. S7 (ESI†) shows that higher Er^3+^ concentrations result in reduced displacement. This effect is likely due to less efficient excitation at 475 nm, caused by the bandgap shift, and is consistent with the lack of propulsion observed for the 20%Er^3+^ sample. The velocities of these MSs were compared with literature values for BiVO_4_-based systems. Although slightly lower, the speeds observed here are notable given that HQ/BQ-propelled BiVO_4_ MSs remain relatively unexplored (Table S2, ESI†).^4,36–38^ Moreover, differences in fuel kinetics and propulsion mechanisms complicate a direct comparison with H_2_O_2_-driven systems.

**Fig. 5:**
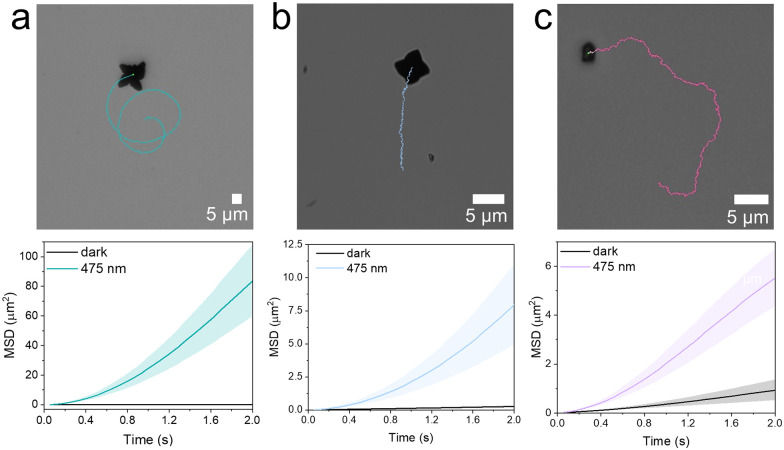
Representative trajectories and MSD of lantanoid-doped BiVO_4_ MSs under 475 nm excitation. (a) Pristine, (b) 15%Er^3+^ and (c) 18%Er^3+^.

To further investigate the reduced motion observed in highly doped samples, we evaluated their photocatalytic activity *via* Rhodamine 6G (R6G) degradation in the presence of H_2_O_2_. After 30 min of illumination, both the pristine and 15%Er^3+^-doped BiVO_4_ MSs exhibited a complete decomposition of R6G. In contrast, the 18%Er^3+^-doped BiVO_4_ MSs showed reduced activity, achieving a 65% degradation, while the 20%Er^3+^ sample was the least effective, reaching only 58% of degradation under the same conditions (Fig. S8 and S9, ESI†). This trend mirrors the previous propulsive behavior observed for the MSs and suggests that higher doping levels not only shift the bandgap but may also introduce recombination centers, both of which contribute to reduced photocatalytic efficiency and impaired visible-light-driven propulsion.

In conclusion, we present a new class of lanthanoid-doped BiVO_4_ MSs prepared using a one-step synthesis procedure that uniquely combine upconversion emission and visible-light-driven propulsion within a single material. By doping BiVO_4_ with Yb^3+^/Er^3+^ through a simple one-step synthesis, we achieved tunable control over morphology, bandgap, and optical response. While higher dopant levels led to mixed crystal phases and increased bandgaps, efficient propulsion under 475 nm light was maintained up to 18%Er^3+^. Simultaneously, UC emission under 980 nm excitation confirmed successful lanthanoid incorporation, enabling direct optical observation *via* fluorescence microscopy. To the best of our knowledge, this is the first report of a single material system exhibiting both UC emission and light-driven self-propulsion, offering new possibilities for the use of multifunctional photoactive MSs in sensing, bioimaging, and environmental remediation.

This work was funded by the European Union (ERC, PhotoSwim, 101076680). Views and opinions expressed are however those of the author(s) only and do not necessarily reflect those of the European Union or the European Research Council. Neither the European Union nor the granting authority can be held responsible for them. This research has also received funding from a 2023 Leonardo Grant (LEO23-2-10594-CBB-QUI-106, RobotsFun) for Researchers and Cultural Creators, BBVA Foundation. J. M. G. thanks the European Union's Horizon Europe research and innovation programme under the MSCA Grant Agreement no. 101148668. Y. C. acknowledges the support from “Juan de la Cierva Grant” JDC2023-051508-I, funded by MICIU/AEI/10.13039/501100011033. L. N. C. U. acknowledges the IVORI master's programme. ICIQ is supported by the Ministerio de Ciencia e Innovación (MICIU/AEI/10.13039/501100011033) through the Severo Ochoa Excellence Accreditation CEX2024-001469-S; and by the CERCA Programme/Generalitat de Catalunya.

## Conflicts of interest

There are no conflicts to declare.

## Supplementary Material

CC-061-D5CC02091C-s001

CC-061-D5CC02091C-s002

CC-061-D5CC02091C-s003

CC-061-D5CC02091C-s004

## Data Availability

The data supporting this article have been included as part of the ESI.†
