# Supplementary material for: Lanthanoid-doped BiVO4 microswimmers with built-in photon upconversion and light-driven motion
Source: Chem Commun (Camb). 2025 Jul 8;61(63):11834–7. doi: 10.1039/d5cc02091c (PMC12235596; doi:10.1039/d5cc02091c)
Supplement: CC-061-D5CC02091C-s001 [file CC-061-D5CC02091C-s001.pdf]

Supplementary Information (SI) for ChemComm.
This journal is © The Royal Society of Chemistry 2025

Supporting Information

Lanthanoid-doped $BiVO_4$ microswimmers with Built-In Photon Upconversion and Light-Driven Motion

João Marcos Gonçalves,[a] Luisa Natalia Cordoba Urresti,[a,b] Yufen Chen [a] and Katherine Villa [a]*

a. Institute of Chemical Research of Catalonia (ICIQ), The Barcelona Institute of Science and Technology (BIST), Av. Països Catalans, 16, Tarragona E-43007, Spain.

b. Departament de Química Física i Inorgànica, Universitat Rovira i Virgili, 43007 Tarragona, Spain

*kvilla@iciq.es

Materials

Rare earth oxides (Y_2O_3, Yb_2O_3 and Er_2O_3, 99.9%) were purchased from ABCR GmbH (Germany), and dissolved in HNO_3 (65%, Chem-Lab, Belgium) to obtain their respective nitrates. All other reagents were used without further modification. Bismuth nitrate pentahydrate ($Bi(NO_3)\cdot 5H_2O$, 99.999%), sodium metavanadate ($NaVO_3$, anhydrous, 99.9%) were purchased from Sigma Aldrich (USA). Potassium chloride was purchased from PanReac Química (Spain). Ethanolamine was purchased from Glentham Life Sciences (UK). Hydroquinone (99.5%) was purchased from Thermo Scientific (USA), and benzoquinone (reagent grade ≥ 98%) was purchased from Sigma Aldrich (USA).

Methods

Synthesis of pristine and Yb,Er-doped $BiVO_4$ microswimmers

The synthesis of the pristine star-shaped- $BiVO_4$ MSs was previously described by Meng [1,2]. First, $Bi(NO_3)_3$ (1 mmol) was dissolved in 30 mL of H_2O, forming a cloudy white suspension. Then, KCl (3 mmol) was added and stirred for 5 minutes, followed by addition of $NaVO_3$ (1 mmol), when the color changed to orange. The pH was adjusted to 2.0 with ethanolamine, and this suspension was stirred for 1 h (37 kHz, 580 W) and then ultrasonicated for an additional hour. Finally, the dispersion was transferred to a 50 mL Teflon flask, and subjected to hydrothermal treatment at 160 °C for 12 hours. The obtained material was filtered, washed with water and ethanol and dried at 70 °C overnight.

The doping was achieved only by adjusting the stoichiometric amounts of $Bi(NO_3)_3$, $Yb(NO_3)_3$ and $Er(NO_3)_3$ solutions. The calculated yields for all samples are given in Table S3.

Characterization of the as-synthesized $BiVO_4$-based microswimmers

Field emission scanning electron microscopy (FESEM) was performed in a Scios 2 (FEI), using a focus Ga ion beam. Electronic Scanning Electron Microscopy (ESEM) and energy dispersive X-ray spectroscopy (EDS) mapping were performed in a Quanta 600 microscope (FEI). Samples were dispersed in ethanol and deposited in silicon wafers. X-Ray diffraction (XRD) was performed in a D8-DISCOVER (Bruker) from 5 to 80 ° at a 10 $°\cdot minute^{-1}$ scan speed. A UV-2401PC spectrophotometer (Shimadzu) equipped with an integrating sphere was utilized for diffuse reflectance.

Emission spectra were recorded using an OmniFluo 900 spectrofluorimeter (Zolix) using a 980 nm laser as excitation source (CNI Lasers) equipped with a collimator with a 1 mm beam diameter at 5 cm distance (maximum power density = 66.2 $W\cdot cm^{-2}$). For fluorescence imaging and motion analysis, a THUNDER Imager DMi8 inverted microscope was used (Leica) using 40x or 100x objectives. Videos were recorded in time-lapse mode at 29 frames per second (FPS), and the microswimmers were tracked for 25 seconds using a freshly prepared mixture of hydroquinone and benzoquinone at a final concentration of 10 mM:5mM proportion. The excitation was performed with a 475 nm LED passing through the objective, giving a power density of 5 $W\cdot cm^{-2}$ for the 40x objective (used only for the pristine MS) and 4 $W\cdot cm^{-2}$ for the 100x objective.

Degradation of Rhodamine 6G

A solution of 10 ppm of Rhodamine 6G was used for the degradation experiments. A suspension of 5 mg of the microswimmers was prepared in 10 mL of this solution and H_2O_2 (30% v/v) was added to a final concentration of 1%.

Photocatalytic degradation was performed illuminating this suspension with a 300 W Xenon light source (ASAHI Spectra) with a 400 nm long pass filter. Spectra were recorded in a UV-1800 spectrophotometer (Shimadzu).

Propulsion mechanism

Asymmetric generation of chemical species is essential for achieving self-propulsion in microswimmers. For the lanthanoid-doped $BiVO_4$ MSs, this asymmetry is likely enhanced by the surface irregularities and heterogeneous roughness, clearly visible in Figure 1.

The propulsion mechanism is most plausibly attributed to self-diffusiophoresis. Upon visible light irradiation, $BiVO_4$ absorbs photons and generates e^--h^+ pairs, which subsequently react with HQ/BQ redox couple according to the following equations [3,4]:

$$BQ + 2e^- \rightarrow HQ + 2OH^-$$

$$HQ \rightarrow BQ + 2H^+ + 2e^-$$

Due to the differing diffusion rates of the generated ions ($D_{H^+} = 9.311\cdot10^{-5}$ cm^{2}s^{-1} and $D_{OH^-} = 5.273\cdot10^{-5}$ cm^{2}s^{-1}), a local chemical gradient and an associated electric field are established near the particle surface, which drives the motion via phoretic mechanisms.[5]

Table S1. Semi-quantitative XRD analysis showing the phase composition of each microswimmer sample

% of Er^{3+}	Monoclinic	Tetragonal	$ErVO_4$
**0**	100%	0%	0%
**15**	773.%	3.1%	19.6%
**18**	50.2%	31.9%	17.9%
**20**	12.2%	57.1%	30.7%

Table S2. Performance comparison of $BiVO_4$-based microswimmers under visible light excitation.

Microswimmer	Fuel	Excitation	Speed (bodylentgh·s^{-1})	Reference
Four-point star- $BiVO_4$	0.1% H_2O_2	Commercial light in visible region (with UV filter)	≈0.83	[6]
Four-point star- $BiVO_4$	1% H_2O_2	469 nm	1.92	[7]
Butterfly-shaped $BiVO_4$	p-nitrophenol	Commercial light in visible region (with UV filter)	0.38	[8]
Spheroidal $BiVO_4$	0.3% H_2O_2	469 nm	2.01	[9]
Pristine $BiVO_4$ stars	HQ/BQ	475 nm	0.38	This work
$BiVO_4$:20%Yb,15%Er	HQ/BQ	475 nm	0.22	This work
$BiVO_4$:20%Yb,18%Er	HQ/BQ	475 nm	0.85	This work

Table S3. Yield of $BiVO_4$-based microswimmers with varying Er^{3+} doping levels.

Sample	Theoretical mass (mg)	Experimental mass (mg)	Yield (%)
**Pristine**	324.0	292.6	90.3
**15% Er**	310.5	209.6	67.5
**18% Er**	309.2	262.5	84.9
**20% Er**	308.4	267.8	86.9

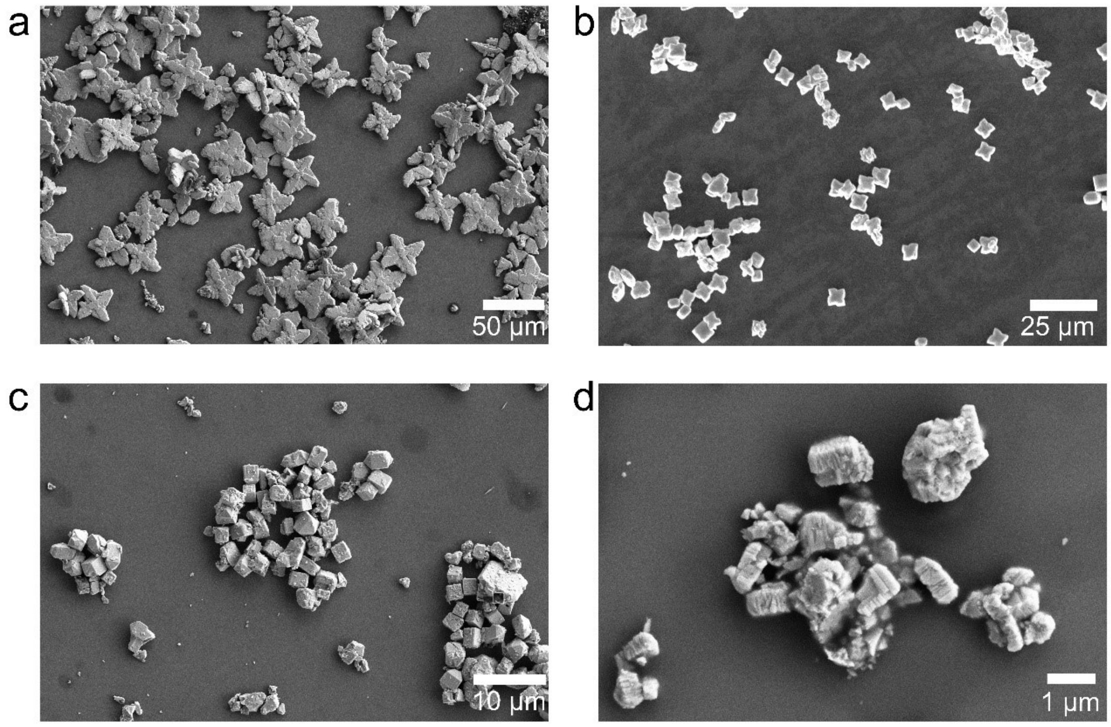

Fig. S1. Low magnification FESEM images of (a) pristine $BiVO_4$, (b) 15%Er, (c) 18%Er and (d) 20%Er.

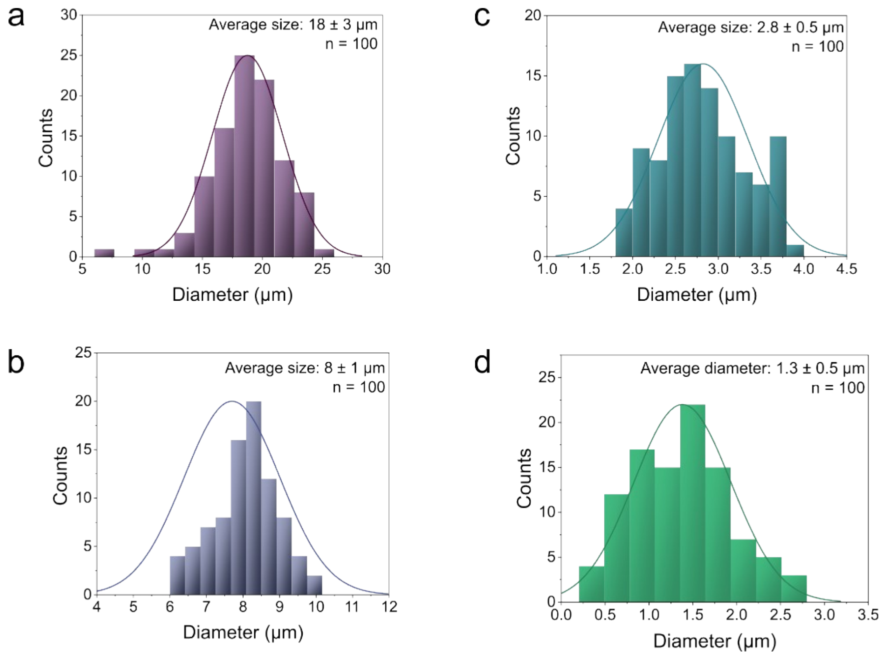

Fig. S2. Size distribution of (a) pristine $BiVO_4$, (b) 15%Er^{3+}, (c) 18%Er^{3+} and (d) 20%Er^{3+}.

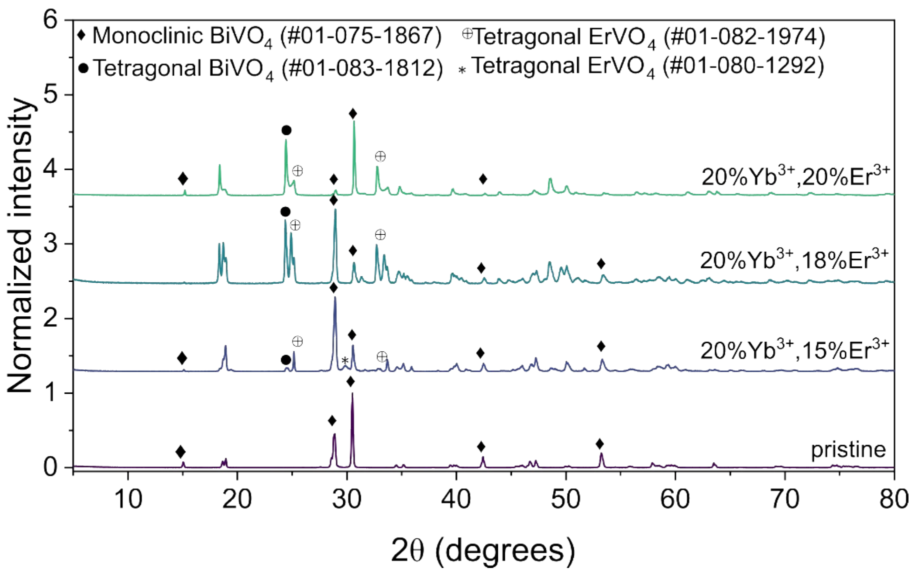

Fig. S3. XRD of the MSs, highlighting some peaks from crystalline phases present.

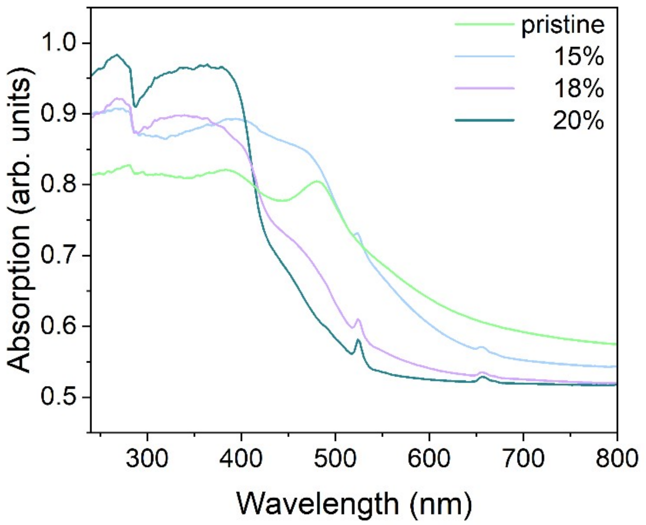

Fig. S4. Absorption spectra of the MSs showing absorption bands attributed to Er^{3+}.

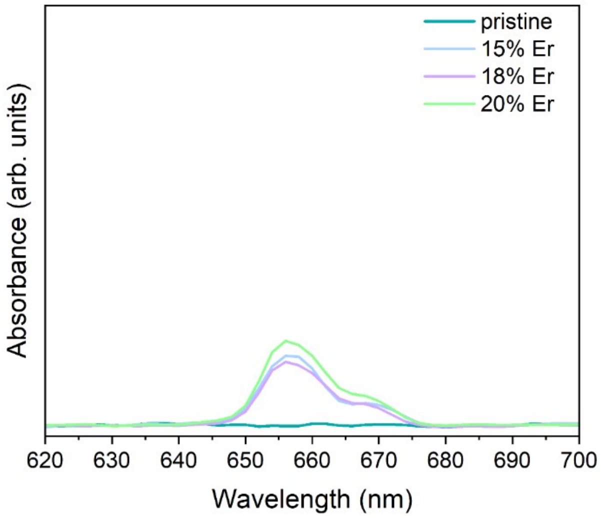

Fig. S5. Baseline corrected absorption spectra of the MSs highlighting $^4F_{9/2} \leftarrow {}^4I_{15/2}$ transition from Er^{3+}.

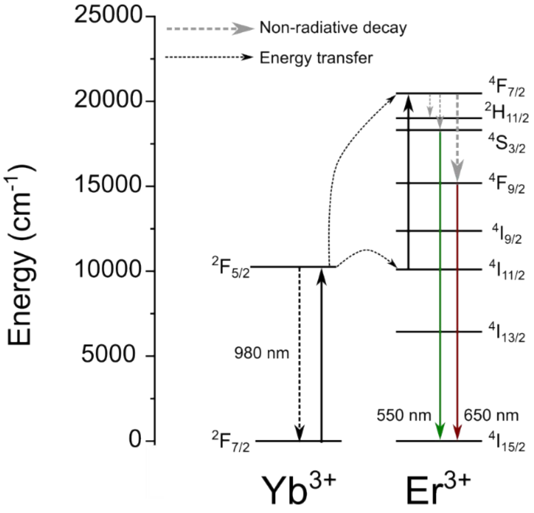

Fig. S6. Energy level diagram illustrating the so-called Energy transfer upconversion (ETU) mechanism for Yb^{3+}-Er^{3+} doped materials.

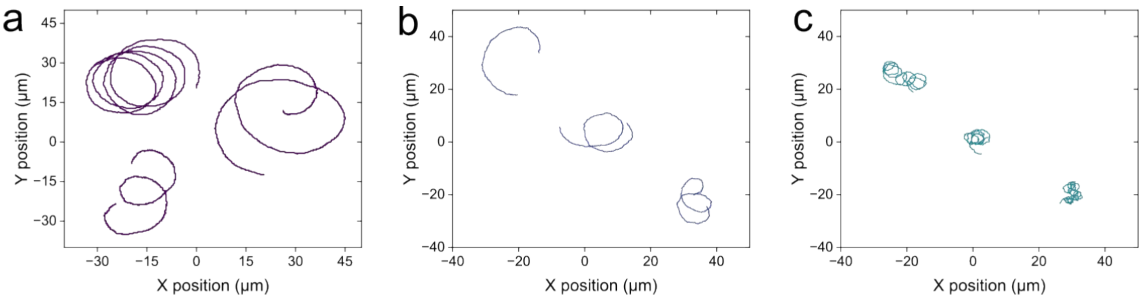

Fig. S7. Representative trajectories of the MSs under 475 nm illumination. (a) pristine (power density = 5 W·cm^{-2}), (b) 15%Er (power density = 4 W·cm^{-2}) and (c) 18%Er (power density = 4 W·cm^{-2}).

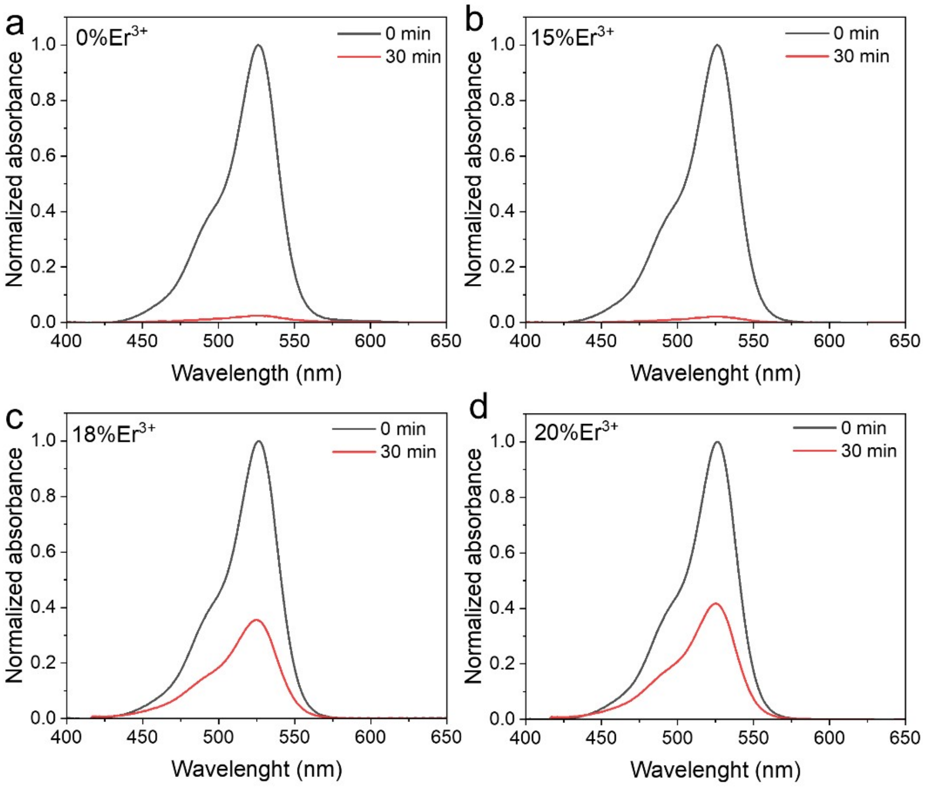

Fig. S8. Normalized absorption spectra of the degradation of Rhodamine 6G by the MSs in 1% H_2O_2 after 30 minutes. (a) pristine, (b) 15%Er^{3+}, (c) 18%Er^{3+} and (d) 20%Er^{3+}.

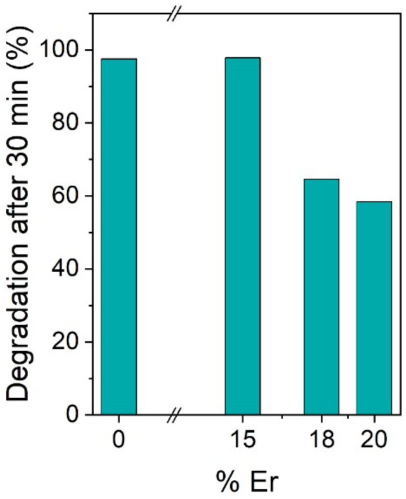

Fig. S9. Rhodamine 6G degradation percentage after 30 minutes of light exposition of the MSs in 1% of H_2O_2.

References

1 S. Meng, T. Ogawa, H. Okumura and K. N. Ishihara, *J. Solid State Chem.*, 2021, **302**, 122291.

2 S. Meng, T. Ogawa, K. N. Ishihara and H. Okumura, *Energies*, 2023, **16**, 629.

3 B. Dai, J. Wang, Z. Xiong, X. Zhan, W. Dai, C. C. Li, S. P. Feng and J. Tang, *Nat. Nanotechnol.*, 2016, **11**, 1087–1092.

4 M. Cheng, X. Yang, F. Zhang, J. Zhao and L. Sun, *Angew. Chem. Int. Ed.*, 2012, **51**, 9896–9899.

5 D. Kagan, S. Balasubramanian and J. Wang, *Angew. Chem. Int. Ed.*, 2011, **50**, 503–506.

6 K. Villa, F. Novotný, J. Zelenka, M. P. Browne, T. Ruml and M. Pumera, *ACS Nano*, 2019, **13**, 8135–8145.

7 S. Heckel and J. Simmchen, *Adv. Intell. Syst.*, 2019, **1**, 1900093.

8 X. Yuan, R. Ferrer-Campos, F. A. Garcés-Pineda and K. Villa, *Small*, 2023, **19**, 2207303.

9 S. Heckel, J. Grauer, M. Semmler, T. Gemming, H. Löwen, B. Liebchen and J. Simmchen, *Langmuir*, 2020, **36**, 12473–12480.
